# What the American Medical Association Thinks of the Electronic Reactions of Abrams

**Published:** 1923-03

**Authors:** Albert Abrams


### WHAT THE AMERICAN MEDICAL ASSOCIATION THINKS OF THE ELECTRONIC REACTIONS OF ABRAMS.

ALBERT ABRAMS, A. M., M. D., LL. D., F. R. M. S.

### FOREWORD

Dr. Albert Abrams of San Francisco is the latest rocket to blaze a somewhat polychromatic course across the firmament of pseudo-medicine. The stick will fall, anon. In the field of diagnosis Dr. Abrams claims to have evolved a system of abdominal percussion, practiced in connection with certain electrical apparatus he has made, from which he derives what he is pleased to term the "Electronic Reactions of Abrams" (abbreviated E. R. A.). By means of this system it is claimed that Abrams "can diagnose the sex, race and disease" of a patient he has never seen. All that he needs is a sample of blood from the individual. A few drops of blood, from a person who must be facing west but who may be a thousand miles or more away, are put on a piece of paper and the paper is placed in what Abrams calls his "Dynamizer." This is connected with his "Rheostatic Dynamizer," from which, in turn, wires go to the "Vibratory Rate Rheostat" that is connected with the "Measuring Rheostat!" From the "Measuring Rheostat" comes a wire at the end of which is an electrode which is pressed to the forehead of a healthy individual (the "subject") whose abdomen is being percussed. The "subject" must face west, also.

The whole arrangement is reminiscent of one of Goldberg's inimitable cartoons, depicting a fearful and wonderful device for waking up in the morning or committing suicide or something equally interesting. The nub of the whole matter is, however, that the alleged diagnosis is made by mapping out various areas of resonance and dulness in the "subject" who is being percussed. Dr. Abrams claims to be able to tell by this means whether the individual whose blood is being "tested" is suffering from syphilis, sarcoma, carcinoma, typhoid fever, malaria, gonorrhea or tuberculosis and, if so suffering, where the diseased area is located! He can also diagnose pregnancy by the same method!!

So skilful has Dr. Abrams become in his diagnoses that he can substitute for the drop of blood, the *autograph* of an individual, living or dead, and subject it to his tests and declare whether or not the individual is or was a sufferer from syphilis, etc. He has, in fact, reported the results of subjecting the autograph of Samuel Pepys to his "electronic reactions" and finding that this famous diarist suffered from congenital syphilis; of finding the same for Henry Wadsworth Longfellow and also for Edgar Allen Poe and, in the case of the latter, adding that he also got the "reaction of dipsomania." The autograph (written in 1775) of that stern old moralist Dr. Samuel Johnson gave the "reaction" for acquired syphilis and tuberculosis.

Nor is this all. Dr. Abrams now announces that by his "electronic reactions" he can determine the religion of an individual and, in the latest issue (September, 1922) of his house-organ *Physico-Clinical Medicine* he maps out the areas of dulness for (1) Catholic, (2) Methodist, (3) Seventh Day Adventists, (4) Theosophist, (5) Protestant, (6) Jew. Just why the area of dulness of a "Methodist" should be in the left lower quadrant of the abdomen while the area of dulness of a "Protestant" is in the right lower quadrant, is not quite clear. Neither is it explained why the Jew should have such a much larger area of dulness than the Christian. These points will, doubtless, be cleared up later.

In the field of treatment Albert Abrams claims equal marvels. He has discovered that all drugs that are specific in the treatment of disease have a definite vibration rate. He has, therefore, devised another instrument which he calls the "Oscilloclast." This is capable, so it is claimed, of producing vibrations of various rapidities. Instead of using a drug, one starts the "Oscilloclast" going, moves the indicator to the number corresponding to the vibration rate of the indicated drug and applies the instrument to the sufferer, who then gets, it is alleged, the therapeutic action of the drug in question. The "Oscilloclast" is not for sale; it can be leased, to those who are willing to pay the price and sign a contract that they will not open it!

It has been suggested that the American Medical Association conduct a scientific investigation of Abrams' claims. As has been stated elsewhere, Abrams was given an opportunity a few years ago to subject his theories to scientific test—and he rejected the opportunity. To make such an investigation today would be to dignify that which has become preposterous on its face. Nor is it conceivable that an adverse report from a group of scientific men would serve any good purpose. Certainly it would make no difference to the deluded, ignorant or itching palms in the profession who are "enjoying incomes of from $1,000 to $2,000 a week" from the use of the Abrams paraphernalia; while Abrams' own retort would doubtless be that the diagnostic methods of scientific medicine are too crude to be used to check his ultra-refined "electronic reactions." In fact, in warning his disciples not to submit to blood tests, Abrams declared recently (September, 1922) that "the E. R. A. can not fully accord with the conventional diagnoses." The American Medical Association will take up Abrams' fantastic vagaries for serious investigation when the American Astronomical Society appoints a committee to determine the truth or falsity of the theory of Voliva, heal of the Zionists, that the earth is flat.

### "SPONDYLOTHERAPY," "ELECTRONIC REACTIONS," THE "OSCILLOCLAST," THE "ELECTROBIOSCOPE," ETC.

For some time *The Journal* has received inquiries of which the following recent examples are typical. This from an Ohio physician:

"Please give me some information concerning Dr. Abrams and his diagnostic and therapeutic devices known as reflexaphore and oscilloclast. If this is published please withhold my name."

A physician in Massachusetts writes:

"Can you give me any information concerning Dr. (?) San Francisco, California, who reports himself able to diagnose syphilis from a drop of blood sent him on a blotting paper. He has caused a patient of mine a great deal of needless worry."

And from Rhode Island a physician facetiously inquires:

"I am interested to know of the 'Reactions of Abrams.' Have you any information that you can give me in regard to this matter? They apparently do wonderful things in the West."

While a New York physician acknowledges his failure to keep up with the times thus:

"To-day I had occasion to see a patient who mentioned having an Abrams' test for gonorrheal infection of the prostate. He also stated he wished to have Abrams' treatment for the same condition. Could you enlighten me as to what these are? I thought I had kept myself up to date as to all new tests and treatments in my line; but evidently I have been delinquent."

According to our records, Albert Abrams, A. M., M. D., LL. D., F. R. M. S., was born in San Francisco in 1864. He was graduated in medicine by the University of Heidelberg, Germany, in 1882. Dr. Abrams has written voluminously. In 1910, his book on "Spondylotherapy" ("Physio-Therapy of the Spine")[1] was reviewed in *The Journal.*

"Spondylotherapy" is a neologic creation of Dr. Abrams. According to its disciples, it concerns itself "*only* with the excitation of the functional centers of the spinal cord" and has been called "the science of evoking the reflexes of the body both to diagnose and to cure disease." In bringing its review of Abrams' book on "Spondylotherapy" to a close, *The Journal* said: " * * * one wonders whether this is an attempt to explain osteopathy and chiropractic to the understanding of the regular practitioner, or to exploit the very ingenious percussion devices of the author, or whether it is really true that medical men really know practically nothing about the cure of disease through treatment of the spine. Let us hope that it is the latter and that a careful study of this unique volume may open new avenues of therapy heretofore undreamed of."

While the review was obviously critical, yet in advertising the book, the publishers picked out part of the closing sentence, omitted the context, and quoted *The Journal* as having said:

"Let us hope that a careful study of this unique volume may open new avenues of therapy heretofore undreamed of."

When this matter was brought to the attention of Dr. Abrams, he replied, "I fail to see any real difference in the two quotations" and "only one * * * with an astigmatic mentality" could "see any incongruity between the context and the concluding sentence." Yet, in this same letter which attempted to justify the garbling of a quotation so as to make a critical review appear a laudatory one, Dr. Abrams declared that the review in question was "conceived and executed in a malicious spirit."

Between 1912 and 1914 Dr. Abrams gave "clinical courses" on "Spondylotherapy" in various parts of the country—price $50. These "courses" were widely advertised by an Ohio concern that seems to make a specialty not only of handling the advertising campaigns of those members of the medical profession who have unusual or bizarre methods to exploit, but also of acting as an agent for the sale of such devices and publications as may be necessary to the proper practice of the particular brand of therapy that is being exploited. At the time this concern was featuring Abrams' course it called attention to the alleged fact that "no class were [*sic!*] so busy as those employing mechanical treatment such as Osteopathy, Chiropractic, Mechanotherapy, etc."

Says Dr. Abrams:

"Despite the fury of tongue or the truculence of pen, the osteopath and chiropractor are inspiring the confidence of the community with their systems. Right or wrong in their theory, they are, in vulgar parlance, 'delivering the goods.' Spondylotherapy was a product of necessity—the translation of an ignored field of medicine from a chaotic to a scientific basis."

Possibly the following testimonial published by Dr. Abrams as typical of many received, and credited to "Dr. Henry Stacy Dodge, Richmond, Va.," may explain the field that "Spondylotherapy" is to cover. Incidentally, "Dr." Dodge is listed in the Richmond telephone directory as a chiropractor:

"I have been in practice for fifteen years in Chiropractic and ten years an Osteopath and I wish to say that during the last three years, I have received more genuine and sincere satisfaction from the application of Spondylotherapy than all other methods combined. My success in gastrology alone is worth many times the cost of the information."

More recently, Dr. Abrams has advertised that he gives a "course" in Spondylotherapy in San Francisco, beginning on the first of each month. The course lasts four weeks. "The honorarium for this course is $200.00."

In 1912 an organization was created devoted to this new therapeutic method: the "American Association for the Study of Spondylotherapy." Later Dr. Abrams was made Honorary President. Whether the organization is still viable we do not know.

ELECTRONIC REACTIONS OF ABRAMS

In addition to "Spondylotherapy," Dr. Abrams has also evolved what he calls the "Electronic Reactions of Abrams." These are said to make possible long-distance diagnoses, it being necessary only to send a few drops of blood taken from the patient and allowed to dry on a slide. There are, it seems, certain instruments and devices used in the performance of these diagnostic feats. By means of the "Electronic Reactions" Dr. Abrams (while admitting the protective factor of vaccination against smallpox) has discovered that practically all the vaccines obtained from reliable firms yield the reaction ("electronic tests") of congenital syphilis, and that many of them also yield the reaction of tuberculosis and of streptococci and staphylococci. Further, "from the cicatrices of all vaccinated persons, one can always elicit a reaction of congenital syphilis and in early scars a tuberculous reaction." Dr. Abrams also declares that exposing vaccine virus for ten minutes to blue light will destroy the syphilitic, streptococci and staphylococci reactions and exposing it for the same period to yellow light will destroy the tuberculous reaction.

One of Dr. Abrams' disciples—Sir James Barr—frequently quoted with evident satisfaction, declares that from a fresh sample of blood spread over four square inches of white blotting paper, "Dr. Abrams can diagnose the sex, race and disease of the patient." However, there are certain precautions that must be taken: The patient should face West, "the blood should be taken in a subdued light and there should be no strong red or yellow coloring material in the room."

In various places Dr. Abrams has asserted that "if splanchno-diagnosis is approached with a prejudiced mind, it is better not to attempt it, for there are 'none so blind as those that will not see.' "

Dr. Abrams founded and edits *Physico-Clinical Medicine.* It is published by "Physico-Clinical Co." at 2135 Sacramento St., San Francisco—the address, according to the telephone directory, of Dr. Abrams' residence. It is a quarterly "Devoted to the Study of the Electronic Reactions of Abrams and the Visceral Reflexes of Abrams, in the Diagnosis, Treatment and Pathology of Disease." Single copies, one dollar; by the year, two dollars. The publication is, apparently, not entered as second class matter, in fact, presumably, it could not be, as it seems obviously to be an advertising affair. Each issue contains material dealing with "Spondylotherapy," "Splanchno-Diagnosis," "Electronic Reactions" and other discoveries and theories of Dr. Abrams. In it also is published a list of "Some recent visitors at Dr. Abrams' laboratory," the names and addresses of the "Lessees of Oscilloclast" (about which more later) testimonials for Dr. Abrams, etc.

Of course, it carries advertisements of Dr. Abrams' "Physico-Clinical Laboratory" (also at 2135 Sacramento St.) and his "Practical Courses in Spondylotherapy and Electronic Diagnosis and Treatment" ($200 in advance). Some of the devices of Dr. Abrams are also advertised. "No apparatus sold on credit. Terms cash." Among these are:

"Dr. Abrams' Electrodes for Electronic Diagnosis, $6.00; Biodynamometer, $36.00; Dr. Abrams' Reflex Set, $36.00; Dr. Abrams' Electro-Concusser, $120.00."—From the *Journal of the A. M. A.*, March 25, 1922.

Our apparatus is leased only to physicians who have taken our course. The cost of the apparatus is approximately $400.00. Cost of tuition for the course is $300.00, which includes a complete practical course in personal instruction, which has not heretofore been given. The course lasts one month, and is open to M. D's. and Osteopaths only.

